# O.1.2-3 Engaging middle aged and older adults to participate in physical activity interventions: a qualitative study into persuasive communication messages

**DOI:** 10.1093/eurpub/ckad133.090

**Published:** 2023-09-11

**Authors:** Janet Boekhout, Rieteke Hut, Denise Peels

**Affiliations:** Open University of the Netherlands, Netherlands; janet.boekhout@ou.nl; Open University of the Netherlands, Netherlands; janet.boekhout@ou.nl; Open University of the Netherlands, Netherlands; janet.boekhout@ou.nl

## Abstract

**Purpose:**

Among middle aged and older adults (>50 years) (MAOA) insufficient PA is highly prevalent. Although many PA interventions for MAOA are effective in research settings, the actual impact on public health is often limited, due to low participation when implemented in real-life settings. The issue of low participation in PA interventions is well-documented, but most research so far seems to focus on demographic characteristics as potential explanations, rather than explanations that may lie within the actual communication about the intervention. Therefore, more insight in how persuasive communication can be applied when implementing PA interventions is relevant. This study examines the opinions of MAOA on how communication about PA interventions should be shaped.

**Methods:**

We conducted 31 semi-structured interviews with MAOA. Recruitment was done by way of convenience sample, within the personal and professional network of the researchers. Thematic analysis was applied by using a-priori defined themes based on the persuasive communication theory of McGuire, i.e. the message, channel, source, receiver and target-behavior.

**Results:**

Preliminary findings demonstrated that the message content, channel, and source should be aligned with certain demographics of the receiver. For example, the message should focus on social and mental benefits on PA, rather than referring to age-associated limitations or health benefits. In contrast to middle aged adults, the older adults seemed to dislike social media as channel. General practitioners were the preferred source to inform about interventions. Regarding the target behavior, words like “sports” should be avoided in favor of words like ‘being active’. Final results will be available within 3 months.

**Conclusions:**

By identifying the features that MAOA prefer regarding the message, channel, source, receiver and target behavior of communication about PA intervention, relevant insights about how MAOA can be reached for interventions will be gained. This can contribute to moving beyond merely developing PA interventions that are proven effective in research settings, to implementing such interventions in a way that contributes to reaching sufficient numbers of MOAO to participate in PA interventions, thus substantially contributing to public health.

**Support/Funding Source:**

This research was funded by ZonMw Implementation network Sports and PA.

